# Pulmonary Vein Isolation with a Multielectrode Basket Catheter

**Published:** 2007-04-01

**Authors:** Takumi Yamada

**Affiliations:** Division of Cardiovascular Diseases, University of Alabama at Birmingham, Birmingham, AL, USA

**Keywords:** atrial fibrillation, pulmonary vein isolation, multielectrode basket catheter, catheter ablation

## Abstract

Pulmonary vein (PV) isolation (PVI) techniques have evolved as a curative treatment of atrial fibrillation (AF) since PVI guided by circumferential mapping with a circular catheter was initially proposed. A multielectrode basket catheter (MBC) is also useful for PVI because of some advantages; (1) an MBC provides some information about the PV anatomy on the fluoroscopic image, (2) an MBC can utilize the non-fluoroscopic navigation system, (3) an MBC enables the direct three-dimensional mapping around the PV ostium and antrum, (4) the distal electrodes of the MBC can be used to monitor some activation changes within the PV in real time and thereby indicate the effects of ablation at the ostium and antrum as radiofrequency lesions are created. PVI with an MBC is an effective and safe procedure to cure AF by integrating the PV anatomy and electrophysiology in combination with a non-fluoroscopic three-dimensional navigation system for the ablation catheter.

## Introduction

The pulmonary veins (PVs) have been demonstrated to be the major source of triggers of atrial fibrillation (AF) [1]. Since PV isolation (PVI) guided by circumferential mapping with a circular catheter was initially proposed by Haissaguerre et al. [2], PVI techniques have evolved to improve the clinical results. Because PVI with a circular catheter has several disadvantages and a relatively low success rate [2], some PVI techniques with other mapping tools have been proposed [3-5]. In this review article, we described the technique, efficacy and safety of the PVI with a multielectrode basket catheter (MBC) and its situation in the current catheter ablation of AF.

## The multielectrode basket catheter and mapping system

### Basket catheter and sheath

**Figure 1:**
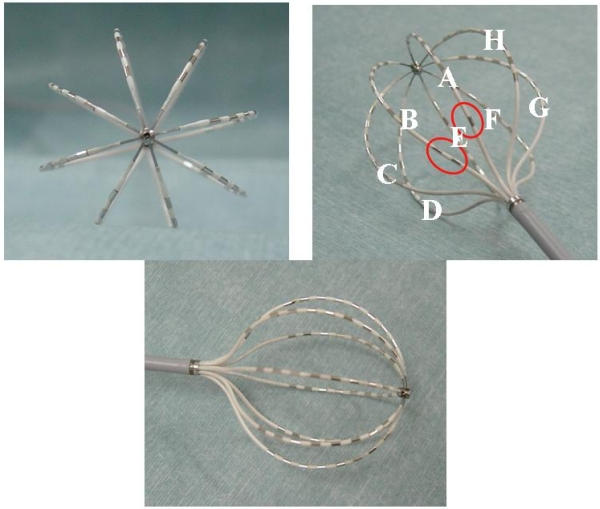
A multielectrode basket catheter (MBC). With a 31-mm MBC, for orientation, spline A or B can be distinguished by one or two radiopaque markers (red circles) on the proximal portion of the spline, respectively.

The MBC (Constellation™, EP Technologies, Boston Scientific corporation/ San Jose, CA, USA) consisted of eight flexible, self-expanding splines (A-H) with eight 1-mm electrodes with 2 or 3-mm (31 or 38-mm MBC) spacing (Figure 1). A special guiding sheath (8.5-French, Soft Tip EP Sheath™, EP Technologies, Boston Scientific corporation/ San Jose, CA, USA) was used for the introduction of the MBC.

### Three-dimensional mapping system

A computerized three-dimensional (3-D) mapping system (QMS2™) could construct a 3-D color isochronal or isopotential map from a total of 56 bipolar electrograms recorded by the MBC. The electrical activity in the space between the splines was estimated by a Bicubic-Spline interpolation to construct a continuous map. A color setup with a gradation which corresponded to the relative time or amplitude of the potential could be arranged variously on the QMS map.

### Navigation system

**Figure 2:**
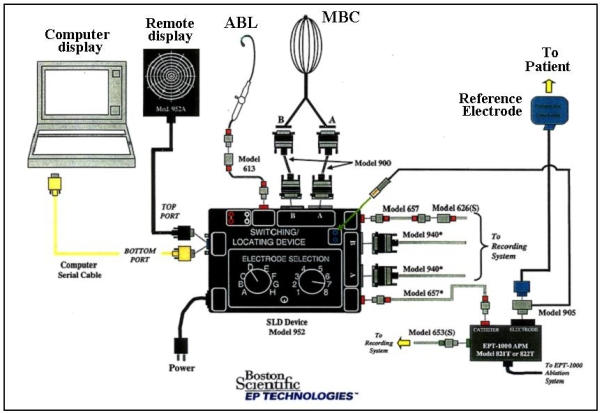
The Astronomer™ system. ABL=the ablation catheter. The other abbreviations are as in figure 1. Reprinted with permission from Boston Scientific Corporation. Same picture without the logo has been published in Yamada T et al. Electrophysiological pulmonary vein antrum isolation with a multielectrode basket catheter is feasible and effective for curing paroxysmal atrial fibrillation: efficacy of minimally extensive pulmonary vein isolation. Heart Rhythm 2006;3:377-384. Copyright (2006), with permission from The Heart Rhythm Society.

A non-fluoroscopic navigation system (Astronomer™) associated with the MBC could be utilized for mapping (Figure 2). This system could show which electrode of the MBC the ablation catheter was positioned at by sensing a weak current from the tip of the ablation catheter via the MBC electrode. This system enabled the ablation catheter to accurately reach the target site indicated by the MBC.

## Deployment of the MBC within the PVs

**Figure 3:**
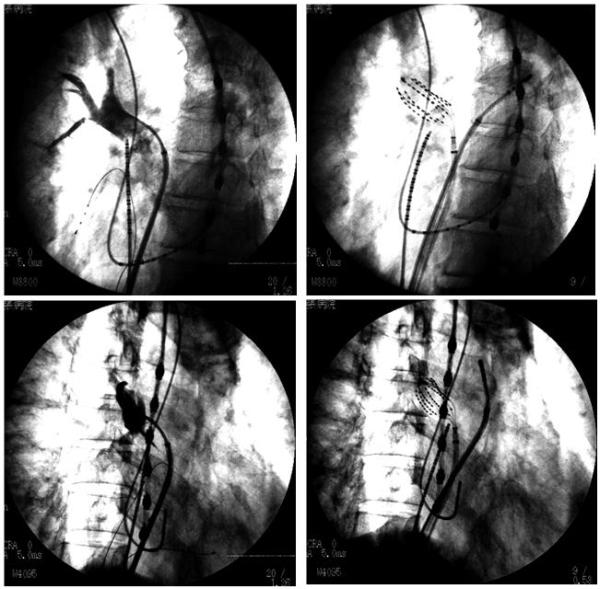
Selective right superior pulmonary vein (PV) angiogram (left panels) and deployment of an MBC within the PV (right panels).The upper panels are in the left anterior oblique (LAO) view and the lower ones in the right anterior oblique (RAO) view.

Prior to the introduction of the MBC within the PVs, a selective PV angiography was performed to evaluate the diameter of the PV and distal branching pattern (Figure 3). A guiding sheath with a 55 degree of curve was most suitable for the superior PVs. However, it was often difficult for that sheath to introduce the MBC into the inferior PVs. Therefore, a guiding sheath with a 120 degree curve was recommended for mapping of all 4 PVs. For the PV mapping and isolation, a 31-mm MBC was usually used. A 38-mm MBC was used for PVs with a dilated diameter or common ostium. The instructions for use recommended that a guiding sheath should be introduced into the distal PVs in advance and pulled back after the MBC was introduced into the sheath until the tip of the MBC reached the tip of the sheath. For introduction into the inferior PVs, the sheath was inserted first with the help of a deflectable ablation catheter [4]. Once the sheath was in place in the PV, the deflectable catheter was removed and replaced by the MBC. The MBC was then deployed in the PV by slowly advancing the MBC while simultaneously withdrawing the sheath. However, our experience demonstrated that it was more feasible for the MBC to be introduced into the PVs directly with the sheath positioned in the left atrium (LA). That technique was never dangerous because the MBC had very flexible splines. Even with that technique, it was sometimes difficult to introduce the MBC into the right inferior PV. In that situation, the technique to slide the MBC below the deflectable ablation catheter positioned at the roof of the right inferior PV and introduce it into the PV was useful. The MBC was deployed within the target PV coaxially to its long axis (Figure 3), otherwise, the MBC would dislodge from the PV easily because of its self-expanding splines. Once the MBC was deployed within the PVs, the self-expanding splines fit the PV wall, which enabled the visualization of the PVs (Figure 3). The instructions for use recommended that intravenous heparin should be administered to maintain an activated clotting time > 300 seconds during the MBC mapping procedure, however, we felt that the recommended activated clotting time level was a little too high for Japanese and possibly other Asian people as well.

## Segmental ostial PVI

Haissaguerre et al. demonstrated that circumferential PV ostial mapping with a circular catheter could identify a segmental electrical connection between the PV and LA and a successful PVI could be achieved by radiofrequency (RF) ablation targeting those electrical disconnections [2]. The MBC was also useful for circumferential PV ostial mapping to identify those electrical connections [4,6-8]. Though a circular catheter was usually placed 10 to 15 mm inside the PVs for the stability of the catheter [2], the MBC was deployed within the target PV with its most proximal electrodes positioned at the PV ostium which was determined by a selective PV angiogram. An RF application was delivered to a preferential electrical connection at the PV ostium identified by the circumferential MBC mapping with the guidance of the Astronomer™ [4,8]. Successful PVI was usually defined as either the abolition or dissociation of the distal PV potentials (entrance block) [4,6-8]. Pacing from the distal electrode pairs of the MBC was useful for confirming the exit block [7].

### Efficacy of the 3-D mapping system

**Figure 4:**
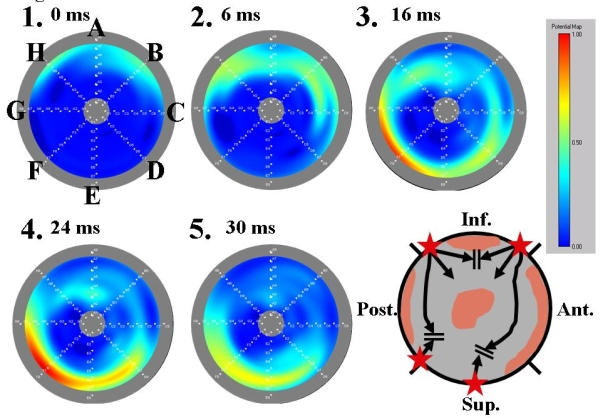
A three-dimensional potential map of the left superior PV (LSPV) with three segmental electrical connections. The gray round outer frame corresponds to the PV ostium and center of the image to the distal PV (bull's eye image). The alphabetical letters from A to H in the figure indicate the MBC splines and the numbers the time order. The rectangular panel shows the color gradation setup. The schema shows the distribution of the PV musculature (gray area), which is identified by the three-dimensional potential map. The black arrow indicates the activation sequence within the PV. The star indicates an electrical connection identified by the three-dimensional potential map. The time intervals between map 1 and the 4 other maps are indicated on the right side of the map numbers. The activation entered into the LSPV through two segmental electrical connections at the inferior (spline B) and posterior wall (spline H) before the ablation. A part of the two activation wavefronts collided at the distal connection of the PV musculature around spline A. Another activation wavefront propagated toward the superior wall and collided with the late activation through the segmental electrical connection at the superior wall (splines E and F).

PV mapping with a 3-D mapping system (QMS2™) was useful for identifying a preferential electrical connection and determining its elimination accurately because it enabled not only a visualization of the activation sequence of the PV potentials but also an adequate evaluation of the activation sequence between the splines [8,9]. Generally speaking, an isochronal map may be useful for analyzing the activation sequence [9]. However, we want to emphasize the superiority of the isopotential map over the isochronal map for the PVI. Both the LA and PV potentials are usually recorded simultaneously around the PV ostium. Therefore, it is essential to minimize the low-amplitude LA potentials and emphasize the high-amplitude PV potentials to construct a clear 3-D map of the PV potentials. In our technique, in principle, the color setup was arranged to assign colors consisting of dark green, yellow and red to the potentials with amplitudes larger than half of the largest amplitude of all the related potentials (color threshold). The serial activation patterns moving around the outer frame of the 3-D PV isopotential map before the longitudinal propagation were defined to indicate the LA-PV junction (Figure 4). The onset of a centrifugal activation on the LA-PV junction was identified as an electrical connection (Figure 4). We demonstrated that our mapping technique using the 3-D PV isopotential map was very effective for segmental ostial PVI because it could automatically provide more useful information for the PVI than an isochronal map [8].

### Comparison with circular PV mapping catheters

**Figure 5:**
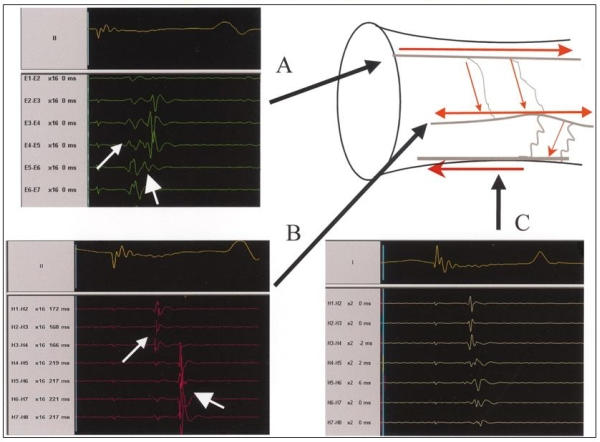
Illustration of longitudinal and transverse patterns of spline activations. A; A spline with longitudinal activation. The earliest PV activity is recorded at electrode pair E 5-6. B; Transverse pattern of activation, manifested as simultaneous recordings along a spline. The simultaneous recording of PV potentials along a spline suggests that the electrodes along the spline are activated by a wide electrical wavefront propagating perpendicular to several electrodes along the spline. C; Transverse activation that is simultaneous on the most distal end (H4-5 through H1-2), but with activation from distal to proximal (from H4-5 through H7-8). When a narrow wavefront traveled initially along the long axis of the vein and later spread perpendicular to the long axis of the vein, it might reach a spline from its distal end first, then travel along a fiber leading to a sequence of activation from distal to proximal. Reprinted with permission from Sanchez, JE et al, Evidence for longitudinal and transverse fiber conduction in human pulmonary veins: relevance for catheter ablation. Circulation 2003; 108: 590-597.

An MBC has several advantages over a circular catheter in PV mapping [4,6-8]. First, an MBC provides some information about the PV anatomy on the fluoroscopic image [4,6-8]. It may be difficult with fluoroscopy alone to be certain just how far a circular catheter is positioned within the PV. This may have important implications for avoidance of PV stenosis [10]. An MBC that conforms to the contours of the LA-PV junction overcomes the limitations of a circular catheter. Second, an MBC can utilize the non-fluoroscopic navigation system [4,6-8]. Third, an MBC enables the direct 3-D mapping around the PV ostium [4,6-8]. The circular catheter records in only one geometric plane. If the LA-PV junction is irregular, it may be impossible to position a circular catheter at the same position relative to the ostium of the PV around its entire circumference. Thus, a circular catheter may record at the LA-PV junction along one side of the catheter while being deeper within the PV along the opposite side. It may not always be possible to position a circular catheter perpendicular to the long axis of the PV. Because of these 2 reasons, it is often difficult to identify a true electrical connection only by circular catheter mapping. However, PV mapping with an MBC can identify the true electrical connections by direct 3-D mapping around the PV ostium, save additional mapping with the ablation catheter and guarantee reproducible mapping. Fourth, the distal electrodes of the MBC can be used to monitor some activation changes in the PV in real time and thereby indicate the effects of ablation at the ostium as RF lesions are created [4,6-8]. In some cases, the elimination of all the PV potentials around the PV ostium observed by circumferential mapping with a circular catheter does not mean successful PVI [11]. The determination of the elimination of all the PVPs is sometimes difficult because the far-field potentials from the left atrial appendage or superior vena cava are superimposed. An MBC that can identify a series of PV potentials from the ostium to the distal PV overcomes the limitation of a circular catheter. Sanchez et al. demonstrated that the mapping of the PV activation with an MBC allowed characterization of the conduction patterns that predicted the requirements for ablation (Figure 5) [6]. The pattern of activation along the splines of an MBC can be used to practical advantage to guide catheter ablation procedures that are designed to achieve PV isolation. When electrical activation within the PV proceeds from proximal to distal along a spline (a longitudinal activation pattern), there is a high probability that ablative energy must be applied at this site in order to achieve an electrical disconnection. In contrast, it is much less likely that ablative energy will be required at sites where evidence of transverse conduction is present. Fifth, an MBC can utilize a computerized 3-D mapping system [8]. In PVI using a circular catheter, the most preferential electrical connection has to be the target of the RF ablation. However, in our technique utilizing QMS2™, intentional PVI according to the manipulation of the ablation catheter is possible independent of the preferential conduction of the electrical connection because the 3-D QMS PV potential map can identify multiple electrical connections simultaneously. Sixth, pacing from the distal electrode pairs of the MBC is available to identify an electrical connection and confirm the exit block during the PVI procedure [7].

On the other hand, an MBC has several disadvantages over a circular catheter [4,6-8]. First, circumferential mapping with an MBC with 8 bipolar electrodes on each of the 8 splines may achieve lower resolution than that of a circular catheter with 10 bipolar electrodes. Second, an MBC non-deflectable catheter requires a special sheath with a limited number of pre-shaped curves. This may present challenges in positioning this catheter within the right inferior PV. Third, the splines may contact one another when the MBC is positioned within a relatively small PV, thereby inducing electrical artifact. Fourth, the splines may not always be equally spaced relative to the circumference of the PV. As a result, areas in which several splines are clustered may be densely mapped while other regions are less densely recorded.

### Clinical outcomes

The AF recurrence rate after the first procedure in the paroxysmal AF patients was 72% and 51% in Kumagai et al.'s and our report, respectively. The AF recurrence rate after multiple procedures was 70 to 80% in the paroxysmal AF patients [4,7,8]. The described advantages of the MBC versus circular mapping catheter-guided PVI2 did not yet result in a better success rate.

The incidence of significant PV stenosis (> 50%) was reduced remarkably (0 to 1.2%) [4,7,8] with an MBC as compared to that with a circular catheter (18%) [10]. The PVI technique with an MBC may minimize the risk of PV stenosis by reducing the number of RF applications and avoiding RF ablation inside the PV.

## Circumferential PVI

Because circumferential PV ablation by an anatomical approach has achieved a better clinical result [3] than segmental ostial PVI [2], 2 circumferential PVI techniques with an MBC were developed [12,13]. Those PVI techniques also aimed at more extensive PV ablation than the previous ones with an MBC in order to cover AF foci outside of the PV ostium in the cause of AF recurrence after segmental ostial PVI [2] and reduce of the risk of PV stenosis.

### PV ostial isolation

Arentz et al. developed circumferential ostial PVI with an MBC [12]. In their technique, the MBC was attempted to be placed so that electrodes 5/6 were at the PV ostium, which was determined by angiography. Circumferential contiguous RF lesions at a distance of > 5 mm from the PV ostia were created using a 7.5-French, 3.5-mm irrigated tip ablation catheter (THERMOCOOL™, Biosense Webster, Diamond Bar, CA, USA) with the guidance of the Astronomer™. For that reason, all positions of proximal electrodes 7/8 from A to H given by the navigation system were targeted by the ablation catheter. When the LA-PV conduction persisted after the circumferential ablation, an electrophysiologic isolation targeting the residual conduction gap with the guidance of the MBC was performed.

After the circumferential ablation, 49% of the PVs had been successfully isolated. In all the remaining PVs with residual conduction gaps, complete PV electrical disconnection could be achieved by an electrophysiologic isolation technique.

### PV antrum isolation

**Figure 6:**
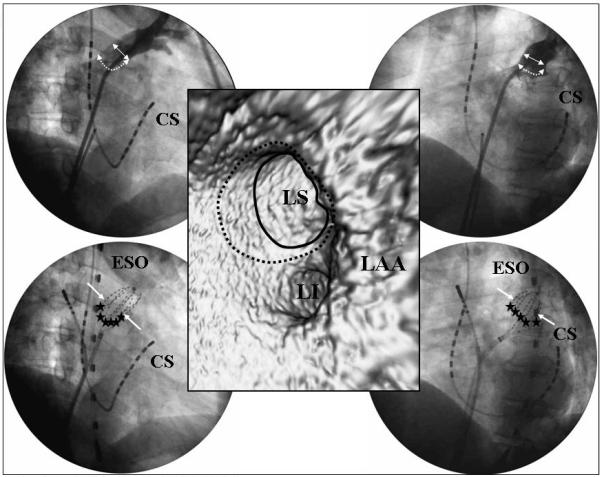
The relationship between the PV antrum and MBC. The upper panels show a selective angiogram of the LSPV and lower panels the position of the MBC. The left panels show the RAO view and right panels the LAO view. The middle panel shows the virtual endoscopic image of the left PVs. The solid arrows and circle indicate the PV ostium, dotted arrows and circle the PV antrum, and stars the PV antrum ablation sites. CS=coronary sinus, ESO=the transesophageal lead, LAA=left atrial appendage, LI=left inferior PV, LS=left superior PV. Reprinted from Yamada T et al. Electrophysiological pulmonary vein antrum isolation with a multielectrode basket catheter is feasible and effective for curing paroxysmal atrial fibrillation: efficacy of minimally extensive pulmonary vein isolation. Heart Rhythm 2006;3:377-384. Copyright (2006), with permission from The Heart Rhythm Society.

**Figure 7:**
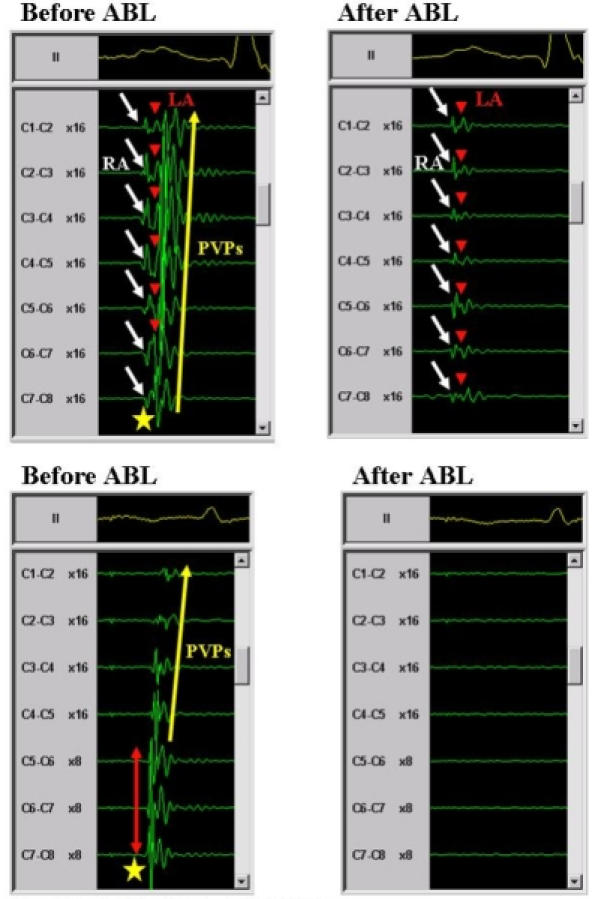
Definition of PV antrum potentials. A upper panels;  An antrum potential (star) recorded from the right superior PV during sinus rhythm (left panel). Note that the antrum potential was a single sharp potential, which was formed by the total fusion of the PV potentials (PVPs) and left atrial (LA) potentials around the PV ostium. After the PV antrum ablation, all the PVPs and PV antrum potentials disappeared and only the right atrial (RA) and LA potentials were observed (right panel). The white arrows indicate the RA potentials, red arrowheads the LA potentials and yellow arrow the PVPs. B lower panels; An antrum potential (star) recorded from the LSPV during distal CS pacing (left panel). Note that the antrum potential had single sharp potentials, which exhibited a transverse activation pattern around the PV ostium. The red arrow indicates the transverse activation pattern and yellow arrow the PVPs. After the PV antrum ablation, all the PVPs and PV antrum potentials disappeared and no potentials were observed (right panel). The abbreviations are as in figure 6. Reprinted from Yamada T et al. Electrophysiological pulmonary vein antrum isolation with a multielectrode basket catheter is feasible and effective for curing paroxysmal atrial fibrillation: efficacy of minimally extensive pulmonary vein isolation. Heart Rhythm 2006;3:377-384. Copyright (2006), with permission from The Heart Rhythm Society.

In general, the PV antrum is defined as the proximal portion next to the tubular PV as observed on the selective PV angiogram or intracardiac echocardiogram [5,13]. It has been reported that PV antrum isolation with the guidance of an intracardiac echocardiogram is very effective in curing AF [5]. We developed a minimally extensive PV ablation technique to isolate the PV antrum with the guidance of the electrophysiologic parameters obtained from an MBC. In our technique, an MBC was introduced toward the distal PV and then pulled back as proximally as possible without dislodgement with fluoroscopic guidance until its most proximal electrodes were positioned at the PV antrum, which was identified by a selective angiography (Figure 6). The MBC was sequentially positioned along the antral circumference when the MBC could not obtain good contact with the antral circumference. A total of 56 bipolar electrograms were recorded by the MBC during sinus rhythm (right PVs) or distal coronary sinus pacing (left PVs). When AF persisted during the electrophysiologic study, cardioversion was used to restore sinus rhythm and an MBC recording of at least one beat was obtained during the appropriate rhythm above. For longitudinal PV mapping using an MBC, we defined the PV antrum potentials as follows: 1) single sharp potentials formed by the total fusion of the PV and LA potentials around the PV ostium (Figure 7A); and 2) single sharp potentials with a transverse activation pattern around the PV ostium (Figure 7B). We defined the transverse activation pattern as the simultaneous activation recorded by some neighboring electrode pairs along the spline. PV antrum ablation was performed using an 8-French, 8-mm tip ablation catheter (Blazer II 5770T™, EP Technologies, Boston Scientific corporation/ San Jose, CA, USA) with the guidance of the Astronomer™ circumferentially targeting the electrode pairs where the PV antrum potentials were recorded with the end-point being their elimination (Figure7). RF applications were also delivered to the gap between the targeted electrode pairs on the neighboring splines in order to produce a continuous RF lesion at the PV antrum. When potentials conforming to the definition of PV antrum potentials were observed from some electrode pairs on the same spline, the antrum potential recorded from the most proximal electrode pair was targeted. If AF persisted during the PV antrum ablation, the RF ablation was performed during AF targeting the electrode pairs where the PV antrum potentials had been recorded during sinus rhythm or coronary sinus pacing after cardioversion. If a residual conduction gap was detected after the PV antrum ablation, additional RF applications to the PV side just next to the previous RF lesions were delivered.

After the circumferential PV antrum ablation, PV antrum isolation was achieved in 77% and a residual PV conduction gap through the previous ablation line was observed in 23% of all the targeted PVs. All of the residual PV antrum conduction gaps were eliminated by a few local RF deliveries and the electrical disconnection of all targeted PV antra was successfully completed.

### Clinical outcomes

The AF recurrence rate after the first procedure was 62% and 84% in Arentz et al.'s (including persistent as well as paroxysmal AF patients) [12] and our report (including paroxysmal AF patients alone) [13], respectively. Circumferential PVI with an MBC could achieve a better result for AF recurrence than segmental ostial PVI with an MBC. In the patients who underwent a second ablation procedure, potential reasons for the AF recurrence were recovery of conduction of previously isolated PVs (89% versus 57%), ostial foci (33% versus 0%), and LA foci (11% versus 29%) after the 2 circumferential PVI procedures, respectively [12,13]. Though 18% of the patients developed LA flutter, which was not previously observed, after the circumferential PV ostial isolation, no spontaneous LA flutter was observed after the circumferential PV antrum isolation. No significant PV stenosis was detected after either of the 2 PVI procedures [12,13].

## Adverse events

Although the splines of the MBC are coated with heparin, the geometry of the MBC is more complex than a circular catheter, a factor that may predispose to greater thrombogenicity. Carbonization can sometimes be observed after ablation on the MBC electrodes or splines. Carbonization is thought to be caused by the concentration of RF energy on the thin splines, which results in very high local temperatures that induce denaturalization of proteins. In spite of those factors, the incidence of thromboembolic events was very low (0.5%) [4,7,8,12,13].

No pericardial tamponade associated with an MBC have been reported in any of the literature [4,6-8,12,13].

## The situation of PVI with an MBC in the current catheter ablation of AF

Over the last decade, considerable advances have been made in the catheter ablation of AF. The success rates have improved with the evolution and development of PV isolation [14,15]. However, more extensive PV ablation may increase the occurrence of residual conduction gaps due to longer ablation lines and a thicker myocardium. The residual conduction gaps can cause not only AF recurrence [15] but also LA flutter [15,16] and the use of the high RF power needed to eliminate those residual conduction gaps may cause critical complications [17]. Therefore, the crucial question has become how extensive the lesions must be to maintain or even increase the success rate while minimizing the risk and complications.

Our electrophysiologic PV antrum isolation with an MBC could eliminate AF without any critical complications in about 90% of the paroxysmal AF patients probably because of achievement of a high occurrence of permanent complete PV electrical disconnection and covering non-PV foci. Therefore, we think that our minimally extensive PV antrum isolation technique can target the optimal sites in order to achieve both a high efficacy and safety. Though our results are preliminary and must be confirmed by further studies with a larger number of patients and longer follow-up, our PV antrum isolation technique seems to be the best of the previous PVIs with an MBC.

The mechanisms of initiating, maintaining, or perpetuating AF are different, complex, and certainly of multiple kinds especially in persistent or permanent AF, and it is reasonable to believe that they cannot be eliminated altogether by PV or PV antrum isolation alone [18]. It was reported that catheter ablation targeting the AF substrate in the LA with the guidance of an electroanatomic mapping system [18,19] improved the clinical results. An MBC is not useful for catheter ablation of the LA AF substrate. However, it has been established that the PVs are the major source of AF [1]. Therefore, it is also important in the catheter ablation of AF, especially paroxysmal, to achieve the maximum effect safely after one procedure of PV ablation. In that sense, our PV antrum isolation technique with an MBC may be very reliable.

## Conclusions

PVI with an MBC is an effective and safe procedure to cure AF patients by integrating the PV anatomy and electrophysiology in combination with a non-fluoroscopic 3-D navigation system for the ablation catheter.

## References

[R1] Haissaguerre M, Jais P, Shah DC (1998). Spontaneous initiation of atrial fibrillation by ectopic beats originating in the pulmonary veins. N England J Med.

[R2] Haissaguerre M, Shah DC, Jais P (2000). Electrophysiological breakthroughs from the left atrium to the pulmonary veins. Circulation.

[R3] Pappone C, Rosanio S, Oreto G (2000). Circumferential radiofrequency ablation of pulmonary vein ostia: a new anatomic approach for curing atrial fibrillation. Circulation.

[R4] Arentz T, von Rosenthal J, Blum T (2003). Feasibility and safety of pulmonary vein isolation using a new mapping and navigation system in patients with refractory atrial fibrillation. Circulation.

[R5] Verma A, Marrouche NF, Natale A (2004). Pulmonary vein antrum isolation: intracardiac echocardiography-guided technique. J Cardiovasc Electrophysiol.

[R6] Sanchez JE, Plumb VJ, Epstein AE (2003). Evidence for longitudinal and transverse fiber conduction in human pulmonary veins: relevance for catheter ablation. Circulation.

[R7] Kumagai K, Ogawa M, Noguchi H (2005). Comparison of 2 mapping strategies for pulmonary vein isolation. Circ J.

[R8] Yamada T, Murakami Y, Muto M (2005). Computerized three-dimensional potential mapping with a multielectrode basket catheter can be useful for pulmonary vein electrical disconnection. J Interv Card Electrophysiol.

[R9] De Ponti R, Tritto M, Lanzotti ME (2004). Computerized high-density mapping of the pulmonary veins: new insights into their electrical activation in patients with atrial fibrillation. Europace.

[R10] Robbins IM, Colvin EV, Doyle TP (1998). Pulmonary vein stenosis after catheter ablation of atrial fibrillation. Circulation.

[R11] Takahashi A, Iesaka Y, Takahashi Y (2002). Electrical connection between pulmonary veins: implication for ostial ablation of pulmonary veins in patients with paroxysmal atrial fibrillation. Circulation.

[R12] Arentz T, Von Rosenthal J, Weber R (2005). Effects of circumferential ostial radiofrequency lesions on pulmonary vein activation recorded with a multipolar basket catheter. J Cardiovasc Electrophysiol.

[R13] Yamada T, Murakami Y, Okada T (2006). Electrophysiological pulmonary vein antrum isolation with a multielectrode basket catheter is feasible and effective for curing paroxysmal atrial fibrillation: efficacy of minimally extensive pulmonary vein isolation. Heart Rhythm.

[R14] Oral H, Christoph S, Chugh A (2003). Catheter ablation for paroxysmal atrial fibrillation: Segmental pulmonary vein ostial ablation versus left atrial ablation. Circulation.

[R15] Ouyang F, Bansch D, Ernst S (2004). Complete isolation of left atrium surrounding the pulmonary veins. New insights from the Double-Lasso technique in paroxysmal atrial fibrillation. Circulation.

[R16] Mesas CE, Pappone C, Lang CCE (2004). Left atrial tachycardia after circumferential pulmonary vein ablation for atrial fibrillation. Electroanatomic characterization and treatment. J Am Coll Cardiol.

[R17] Pappone C, Oral H, Santinelli V (2004). Atrio-esophageal fistula as a complication of percutaneous transcatheter ablation of atrial fibrillation. Circulation.

[R18] Pappone C, Santinelli V, Manguso F (2004). Pulmonary vein denervation enhances long-term benefit after circumferential ablation for paroxysmal atrial fibrillation. Circulation.

[R19] Nademanee K, McKenzie J, Kosar E (2004). A new approach for catheter ablation of atrial fibrillation: mapping of the electrophysiologic substrate. J Am Coll Cardiol.

